# Perceptual Sensitivity to Tonal Alignment in Nuer

**DOI:** 10.1177/00238309231162299

**Published:** 2023-04-25

**Authors:** Siri Gjersøe, Bert Remijsen

**Affiliations:** Institute of Linguistics, University of Leipzig, Germany; Linguistics and English Language, The University of Edinburgh, UK

**Keywords:** Nuer, tone, perception, F0, tonal alignment, JND

## Abstract

This paper examines the perceptual threshold in patterns of tonal timing (alignment) of Falling versus Low tones. The results indicate a remarkable sensitivity among the listeners. In a perception experiment with 30 participants, we tested how native speakers of the West Nilotic language Nuer responded to stimuli in which the timing of the F0 fall that distinguishes Low versus Fall following a High target is manipulated. We measured the threshold for the responses to shift tone perception from 25% to 75%. The results show that listeners only needed an average of 19 ms to differentiate between the melodic shapes and as little as 13 ms for one item. Perceptual sensitivity this fine-grained is not expected based on what is known about the Just Noticeable Difference (JND) from previous studies. Results from non-tonal languages report a sensitivity threshold for tonal timing of at least 50 ms at category boundaries. This difference suggests that whether or not subjects speak a tone language may be a determining factor in their JND.

## 1 Introduction

**Figure 1.**
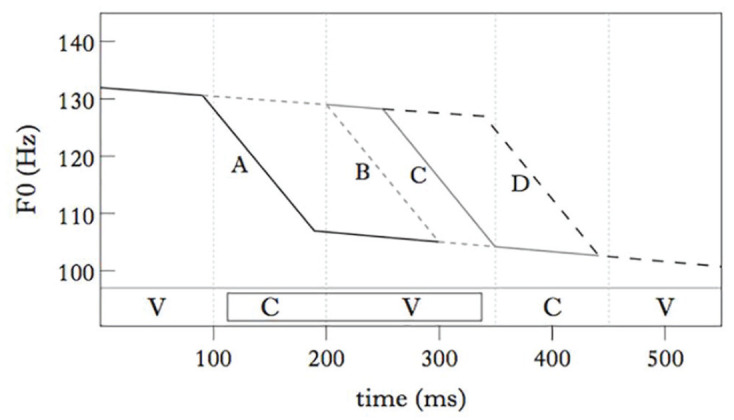
Schematic representations of patterns of tonal alignment. From Remijsen and Ayoker (2014).

A given pattern of fundamental frequency (F0) can give rise to different pitch percepts, depending on how it is aligned with the sequence of consonants and vowels. This is shown schematically by the traces in Figure 1. This figure shows the same F0 shape, that is, a fall from a higher register to a lower register; depending on how this drop is timed within a target syllable domain made up of a consonant (C) and a vowel (V), the resulting auditory percept will be that of a low-pitched syllable in the case of A, but of a high-pitched syllable in the case of D. As for B and C, where the drop in F0 takes place over the vowel—the most salient part of the syllable in acoustic terms—these patterns are prone to be perceived as falling contours.

The important role of F0 alignment in the realization of lexical tone contrasts is well established. In relation to Thai, for example, Zsiga and Nitisaroj (2007) found that the differences between falling versus high tones, and low versus rising tones lie mainly in alignment. Similarly, in Dinka and Shilluk, two languages that are closely related to Nuer, F0 alignment is the primary correlate distinguishing two falling contours, that is, as represented schematically in the contrast between B versus C in Figure 1 (Remijsen, 2013; Remijsen & Ayoker, 2014).

The current study aims to advance our knowledge of this topic by addressing the following question: how discerning are humans in the perception of patterns of tonal alignment? In other words, how close in terms of tonal timing can two F0 patterns be, for example, A and B in Figure 1, and still be reliably distinguished? In this sense, gauging the perceptual sensitivity to tonal timing is essential to a comprehensive account of the way in which perception constrains tonal phonology in the world’s languages.

### 1.1 Just Noticeable Difference and previous studies

There is extensive evidence that the discriminatory ability of speech sounds in general is more sensitive across a category boundary than within the range of phonetic realizations of a speech sound category (Liberman et al., 1957; Schouten & van Hessen, 1992). In a survey of perception studies on patterns of tone and intonation, House (2004) finds that most report a timing sensitivity of at least 50 ms at category boundaries. This threshold of timing sensitivity can be considered to be an estimate of the Just Noticeable Difference (JND) for non-tonal languages.

However, there is evidence that speakers of tone languages perceive F0 patterns in a more fine-grained manner than speakers of non-tonal languages (Gandour & Harshman, 1983; Pfordresher & Brown, 2009) and that the perception of word-level specification for tone is more fine-grained than that of utterance-level or intonational specification for tone (Gussenhoven & van de Ven, 2020). For this reason, it is worthwhile to raise the question of the perceptual sensitivity to tonal alignment in relation to tone languages. It is likely that the JND differs depending on the functional load of tonal alignment, but studies on this topic are skewed: While there are many studies on perception of tonal alignment in non-tonal languages, there are few on tone languages.

To the best of our knowledge, the most fine-grained identification result reported to date is the one in Gårding et al. (1986). They investigated the contrast between the Low and Fall tones of Mandarin (conventionally known as “Tone 3” and “Tone 4”). Following a preceding high tone target and followed by a low target, the Mandarin Low is realized as an F0 fall that takes place during the onset consonant and is followed by low F0.^1^ In contrast, the Mandarin Fall is realized as an F0 fall that starts at the beginning of the vowel. Gårding et al. (1986) created a five-step continuum between these patterns, with a step size of 32 ms, and presented the stimuli in the tonal context of a preceding syllable ending in a high tone target and a following syllable that begins with a low target. The extremes of the continuum are similar to the configurations represented schematically by traces A and B in Figure 1. Mandarin native-speaker subjects were tasked to identify these auditory stimuli as either the Low-toned word or the Fall-toned one. Interpolating on the basis of their results, the perceptual threshold between these tonal categories, that is, the point at which perception is at 50% for either of the two categories, corresponds to a stimulus in which the fall in F0 starts during the onset consonant, 20 ms before the beginning of the vowel. On the basis of the same interpolation, the 50% shift from primarily Low tone responses (75% or more) to primarily Fall tone perceptions (75% or more) took place over a span of just 22 ms (between 31 and 8 ms before the beginning of the vowel).

This finding is remarkable, because other identification experiments investigating such contrasts report substantially greater differences. For example, in a perception experiment with native speakers of Dutch, Verhoeven (1994, p. 76) reports an auditory sensitivity threshold of 70 ms for falling F0 patterns.

Given the relation between discriminability and phonological contrast, our claim is that the big differences in JND can be attributed to the fact that Dutch is not a tone language, whereas Mandarin is. Hence, the 50 ms estimate of JND reported in House (2004) may not be accurate for tone languages like Mandarin and Nuer.

Norwegian represents apparent counterevidence against the interpretation that speakers of tone languages are more sensitive to small differences in tonal timing. Although this language has a lexical tone contrast, the sensitivity to tonal timing, the interval of uncertainty is over 70 ms (Kelly and Smiljanić, 2018, p. 349), comparable to the result reported for Dutch by Verhoeven (1994).^2^ Kelly and Smiljanić (2018, pp. 352–353) attribute this to the involvement of perceptual cues other than tonal timing—specifically the height of the tonal targets.

In addition to the height of the tone targets, various other factors also impact the JND. Several studies have shown that, aside from the alignment of the turning point that defines the beginning of an F0 change, perception is affected by other phonetic properties: F0 height, slope, curvature, and segmental durations (Barnes et al., 2021; D’Imperio, 2000; D’Imperio & House, 1997; Niebuhr, 2003; Niebuhr et al., 2011). For example, Franich (2016) manipulated vowel duration in a perception study investigating the contrast between a falling contour and a high tone in Medumba (Bantu, Cameroon). They found that it has a significant effect on the crossover in perception between the contour and the level tone.

Another parameter to consider concerning JND is the experimental design: in relation to the contrast between Low versus Fall in Mandarin, it may be that the subjects’ accuracy is in fact more sensitive than indicated by the results of Gårding et al. (1986), because of the step size. That is, a step size of 32 ms does not offer a fine-grained resolution, if the listeners’ discriminatory ability is at about 22 ms. It would therefore be insightful to examine such a contrast with a smaller step size. In this study, we will do so, in relation to a contrast in Nuer, a Nilo-Saharan language spoken in South Sudan and Ethiopia. This language presents a contrast between a Low and a Fall that is very similar to the contrast in Mandarin investigated by Gårding et al. (1986).

### 1.2 Nuer language situation and phonology

Nuer is a West Nilotic language within the wider Nilo-Saharan language family (Eberhard et al., 2020). It is spoken in South Sudan and in Ethiopia.

The following phonological properties are relevant to this study. Most Nuer words consist of a single closed syllable. The Nuer phonological inventory is rich in terms of suprasegmental distinctions, with independent contrasts of vowel length, voice quality, and tone. Vowel length is contrastive in a ternary manner; we transcribe short vowels without length diacritic, and mark long and overlong vowels using /ˑ/ and /ː/, respectively—for example, /a, aˑ, aː/. The voice quality contrast distinguishes between modal and breathy voice. As for tone, the inventory varies between dialects. In the Ethiopian variant, the main dialect under investigation here, there is a contrast in underlying specification between H(igh), L(ow), and toneless syllables. The H toneme is realized as a Fall (HL) when the syllable it is associated with has a modal-voiced vowel (cf. Monich, 2020). In the transcriptions and elsewhere in this paper, we are referring to tone in terms of the surface-phonological specification.

### 1.3 L versus HL in Nuer

**Figure 2.**
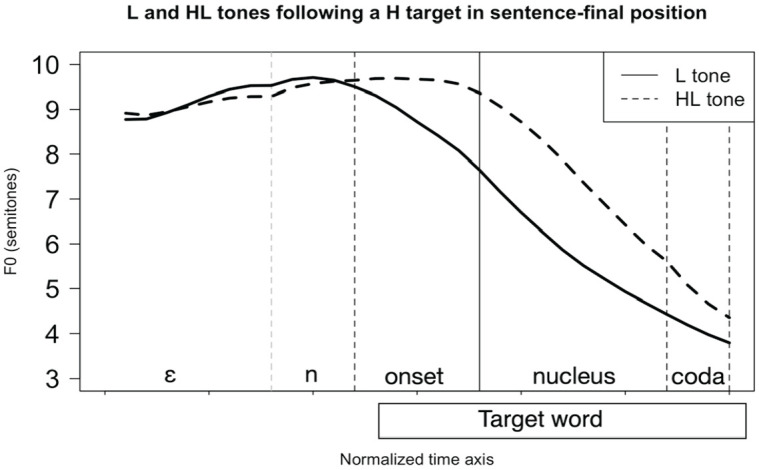
Time-normalized F0 traces plotted from averaged values of L- and HL-toned nouns. The target nouns are pronounced utterance-finally following an H target. The *x*-axis shows the normalized time, and the *y*-axis F0, expressed on the semitone scale.

The phonetic realizations of the L and HL tones in Nuer are illustrated in Figure 2, which is based on data in Gjersøe (2019). This figure shows time-normalized F0 traces for the L and HL tones, averaged across items and speakers. It is based on recordings from six native speakers, who pronounced 10 L-toned items and 12 HL-toned items in sentence-final position in a fixed carrier sentence shown in (1) below.^3^ Here we summarize key results from that study, complemented with additional statistics.

The results in Figure 2 shows that the realizations of the two tone categories are well separated during the vocalic nucleus. The difference between the L tone and the HL tone lies mainly in the timing of the fall in F0 within the syllable domain: the F0 fall starts at the beginning of the onset consonant in the case of the L tone, and at the beginning of the nucleus in the case of the HL tone.

(1) Carrier sentence
  a. nέˑn **l $\overset{ˋ}{ʊ}$ ɲ** see.imp.sg lion “Look at the lion!”
  b. nέˑn  **jâŋ** see.imp.sg cow “Look at the cow!”

We examined the difference in phonetic realization between L versus HL by means of a linear discriminant analysis (LDA). LDA is useful for determining to what extent a given measure distinguishes categories from each other. In this study, we use it to determine to which degree particular F0 measurements can differentiate between L versus HL. An effective measurement is expected to predict a category well above the chance-level baseline, which lies at 50% in this case, as there are two categories involved.

We investigated three different F0 measurements in relation to the monosyllabic target words in the production study in Gjersøe (2019): the F0 value at the temporal midpoint of the vowel (Midpoint); the F0 slope over the vowel (Slope);^4^ and the time point that defines the beginning of the F0 fall (Relative fall onset). The latter measurement is calculated as follows (cf. Remijsen & Ayoker, 2014); Through a script, we first derived a stylized F0 trace. Within this trace, we determined the location of the biggest F0 drop between consecutive points, from halfway into the vowel of the preceding syllable onward up to the end of the vowel of the target syllable. The initial turning point of this F0 fall is interpreted as the start of the fall, and this time point is then expressed relative to the beginning of the vowel of the target syllable.

The LDAs were performed separately with each of these three measurements, with the F0 (Hz) values *z*-transformed by speaker. The results showed that Midpoint and Relative fall onset came out best: Midpoint alone can predict the correct classification of tone with 84.7%, and the Relative fall onset resulted in correct classification in 82.4% of the items. In the case of Slope, in contrast, correct classification was much lower, at 63.3%, which is only 13.3% above the chance-level baseline.

The results from these LDAs inform the choice of measurement to be manipulated in the perception experiment. If the LDAs are considered together with the F0 traces in Figure 2, it is clear that the timing of the fall distinguishes between the L versus HL tone categories substantially better than Slope does. Moreover, by varying the timing of the fall we can control the F0 value over the syllable and its vocalic nucleus. This warrants the choice of the timing of the F0 fall as the phonetic parameter under manipulation in this perception study.

Against the background of earlier work by House (1990, 1996, 1999), it is worthwhile to note that the timing of the initial turning point of the HL tone observed in Nuer (Figure 2) is unexpected. House (1990, pp. 133–134) hypothesized that, to be optimally perceived as a falling contour tone, the turning point that defines the beginning of the falling F0 movement needs to be aligned 30–50 ms into the vowel. This stands in clear contrast to Nuer, where the initial turning point of the HL tone is aligned at the boundary between the onset and the vowel.

## 2 Method

### 2.1 Materials

The material used in the stimuli for the perception experiment consists of three minimal pairs, presenting L versus HL tones. Two pairs have an overlong vowel, and the third has a short vowel. We were not aware of any minimal pair for L versus HL on a long vowel with a sonorant onset, and therefore, could not include all three levels of vowel length. Care was taken to ensure that the onset consonant is sonorant, that is, carrying F0, because this is the segment during which the category boundary between L and HL tones is to be found (cf. Figure 2). In contrast, whether or not the coda is voiced or not is unlikely to be relevant to the phonetic realization of the contrast. They were uttered in sentence-final position following an H-toned word: the imperative verb *nέˑn* “look at.” The stimuli are shown in examples (2)–(4) below. Throughout this paper, we use italics for the phonetic representation in the text.

(2). *Minimal pair maːr*  (3) *Minimal pair laθ*^5^  (4) *Minimal pair wa:r*

  a. nέˑn  mâːr    a. nέˑn  *lâθ* a. nέˑn  wâːr

   see.imp.sg mother \ 1sg  see.imp.sg cotton      see.imp.sg sheep.dung

   “Look at my mother!”   “Look at the cotton!”    “Look at the sheep dung!”

  b. nέˑn màːr     b. nέˑn  *làθ*      b. nέˑn  wàːr

   see.imp.sg relationship  see.imp.sg shaking      see.imp.sg grass

   “Look at the relationship!” “Look at the shaking!”    “Look at the grass!”

**Figure 3.**
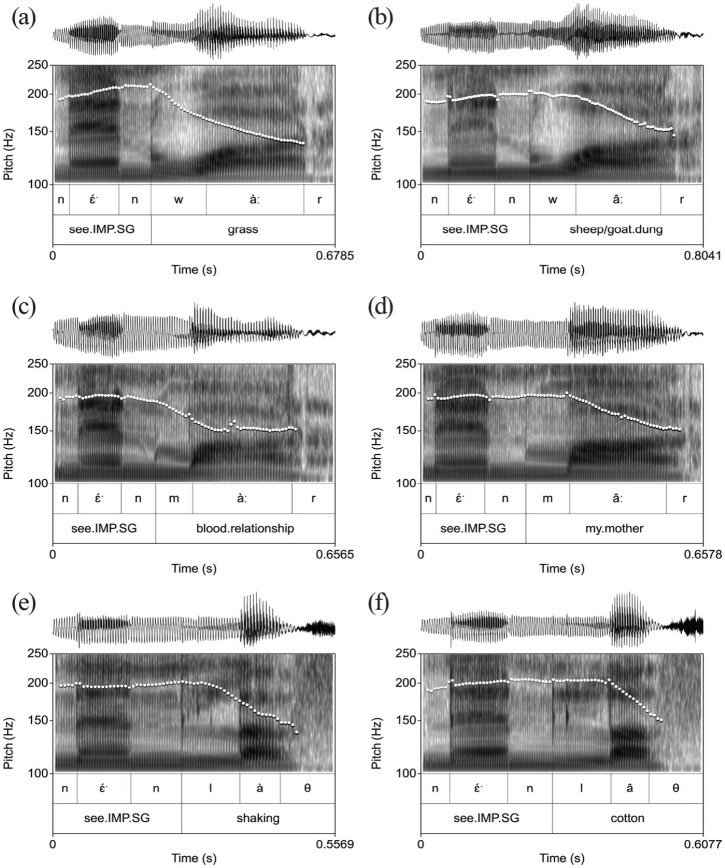
Original tokens of stimuli showing segmental boundaries of each noun. (a) L-toned item, (b) HL-toned item, (c) L-toned item, (d) HL-toned item, (e) L-toned item, and (f) HL-toned item.

The speech data used for the creation of the stimuli in the perception experiment were produced by a 38-year-old female speaker who self-identified as a member of the Gajaak community in Ethiopia. Figure 3 shows the F0 contours of the original tokens prior to manipulation. As these pitch tracks show, the coda is voiceless across all items: [r] or [ θ ]. Note that, in panels (b,d,f) of Figure 3, the turning point that defines the beginning of the fall of the HL tones is aligned exactly with the beginning of the vowel, that is, at the boundary between the onset consonant and the vowel. The boundaries between the vowel and the coda were marked where the F2 energy is critically damped (Turk et al., 2006).

In the creation of the stimuli, our primary concern was ecological validity, that is, for the stimuli to faithfully reflect the realizations by the speaker of the source materials. Hence, the two extremes of the continua of tonal alignment match the production data by the speaker. However, this means that the phonetic parameter slope—which in the source materials is steeper in the short-vowel *la* θ set (Figure 3(e) and (f)) than in the long-vowel *waːr* and *maːr* sets (Figure 3(a) to (d))—differs likewise in steepness between sets in the stimuli. We will return to this in the discussion of the results.

The stimuli were created in Praat on the basis of the HL-tokens of the minimal pairs. The durations of the vowels of the target nouns were 242 ms for the *waːr* set, 224 ms for the *maːr* set, and 90 ms for the *la* θ set. For each of the three sets, we started out from an empty manipulation tier (PitchTier), and then added three F0 points: one at the beginning of the vowel of *nέˑn*, one at the beginning of the target word’s vowel, and one at the end of it. In the manipulations, the first point was kept constant while the two latter were shifted in the alignment manipulations so that the slope would remain constant. The first two F0 points were set at 200 Hz, while the third point at the vowel-coda boundary was set at 150 Hz. The height of these points was kept constant. The fall from 200 to 150 Hz corresponds to approximately 5 ST. The sizes of the F0 falls in *lâ* θ , *wâːr*, and *mâːr* were all between 4 and 5 ST so that a fall of 5 ST captured the natural production of HL tones for the female speaker of this study.^6^

Across the stimuli, the duration of the coda consonant of the verb and of the onset of the target word were set at fixed durations, chosen on the basis of the production data by the speaker: the duration of the coda (/n/) of *nέˑn* was set at 70 ms, and the onset of the target word (/w,m,l/) at 75 ms. These manipulations were carried out in Praat (Boersma & Weenink, 2016) through PSOLA resynthesis.

**Figure 4.**
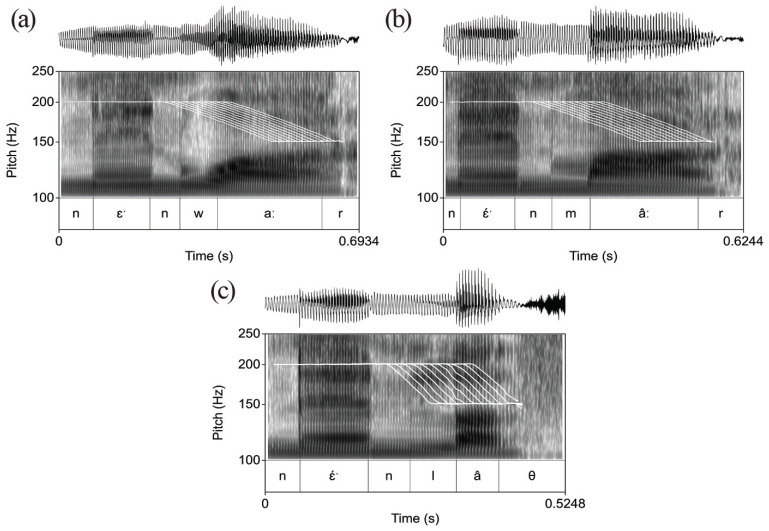
Stimuli for F0 the alignment experiment: 11 manipulation steps of alignment. (a) F0 manipulations of sentence: “Look at the sheep dung!.” (b) F0 manipulations of sentence: “Look at my mother!.” (c) F0 manipulations of sentence: “Look at the cotton.”

The alignment was shifted in steps of 15 ms (see Table 1), relative to the boundary between the onset consonant and the vowel of the target word: eight steps to the left and two steps to the right, resulting in a total of 11 timing patterns for each minimal pair. The resulting F0 contours are shown in Figure 4(a), (b) and (c).

**Table 1.** Manipulation Steps of Stimuli: Timing of the F0 Fall in ms Relative to the Onset-Nucleus Boundary at 0 ms.

Location of F0 peak	Preceding verb			Target noun							
	Coda			Onset					Nucleus		
Steps in ms	−120	−105	−90	−75	−60	−45	−30	−15	0	15	30

### 2.2 Listeners

Thirty-two native speakers of Nuer (30 male, 2 female) took part in the perception experiment. The majority were in their twenties. The youngest participant was aged 21, and the oldest was 56 years old. The speakers came from a continuous geographical area around the border between Ethiopia and South Sudan. The majority of the subjects came from Ethiopia and self-identified as members of the Gajaak and Gajiök communities. In total, the participants self-identified themselves as members of the Gajaak (13), Gajiök (12), Gaguang (3), Lou (3), and Thien (1) communities. Possible dialect differences between these communities have not been studied, but they are taken into account in the preliminary statistical analyses.

Two participants performed at chance level and their results were disregarded. At the early end of the three continua, where an L tone was expected to be perceived (at −120 ms), the two participants still responded just above (55%) or just below (44%) chance level. During the training, it was clear that they had not fully understood the task. The background of these two participants did not stand out from the rest of the participants: They were both male, aged 27 and 31, and self-identified as members of the most common communities (Gajaak and Gajiök) coming from the area of Ethiopia bordering South Sudan. The analyses reported in section 2.4 are based on the remaining 30 participants.

### 2.3 Procedure

The experiment took place in Addis Abeba. The listeners participated in a quiet room, on the premises of one of two universities or of SIL Ethiopia. They were seated in front of a laptop with headphones. The stimuli were presented using the ExperimentMFC tool in Praat. Each stimulus was presented in random order using < PermuteBalancedNoDoublets >, an option that randomizes with the provision that the same stimulus cannot appear twice in a row.

The stimuli were presented in 6 blocks with pauses in between the blocks. Each manipulated token was presented for the listener sequentially. Once the sound file was played, they were asked to choose between the two meanings of the minimal pairs in English: *Look at X* versus *Look at Y*. Using the English translations as opposed to images or descriptive statements or synonyms in Nuer was the fastest and most convenient approach, since all of the participants were proficient in English. English is the third language of the participants, after Nuer and Amharic. Given so, it is highly unlikely the fact that the test was administered using English would have influenced L1 perception. Before the start of the experiment, the nouns were presented with a context to ensure that the word meanings were concrete.

In the experiment, the 11 manipulation steps were repeated three times for each item. This resulted in 99 stimuli token across the three items (sets). The experiment took 7–10 min, depending on how much of a break subjects chose to take. Prior to the experiment, there was a training phase in which a selection of stimuli were presented to familiarize the listeners with the task. This training phase lasted 1–3 min. There was a replay button available on each presentation in case the listeners did not hear the stimulus well the first time. This option, however, was only used by a few participants during the training phase and not during the experiment itself.

### 2.4 Analyses

The analyses aim to determine how the manipulation of F0 alignment affects the perception of L versus HL tones. All statistical analyses were carried out in R (RStudio Team, 2020). First, a multiple correspondence analysis (MCA) was run on the questionnaire information of the participants which had been gathered about their origin to test for the distribution. The tool used for this was MCA in *R* (*FactoMineR* package developed by Lê & Husson, 2008). For studies with many categorical variables, MCA can be used to model these variables on a dimensional space and see their contribution to the variation of the data. The following five variables were modeled in MCA: Community (Gaguang, Gajaak, Gajiök, Lou, and Thien), Dialect of the participant’s Parents (Dinka, Gajiök, Lou, and Pangak, or the same as the participant), Country (Ethiopia, South Sudan, or the border area between Ethiopia and South Sudan), place of Origin (rural area or town), and Sex (male or female).

Then, psychometric curves were plotted in *R* using the package *ggplot2* (Wickham, 2016) to show how the slope of the responses changed for the manipulation steps. To determine the overall effect of the manipulations and other factors on the responses, a polynomial regression model was run with the *glmer* function in *R* (package lme4 version 1.1-12, developed by Bates et al., 2015). The dependent variable was *Response tone* (L or HL). Independent variables were (a) *F0 Alignment*, measured in milliseconds (ms) and (b) *Stimulus* (*waːr, maːr* and *la θ *). In addition, *Listener* was included as a random factor. The polynomial model was used because of the nonlinear relation of the data. It can capture data points which change directions by using several breaking points (“knots”). To determine how many knots are needed in the regression, a model comparison in ANOVA was done. The outcome was that four knots are required. In addition, the MCA allowed for an optimized way to test the significance of the categorical variables. The outcome of the MCA, the contribution of the categorical variables to *Dimension 1*, was tested for relevance by including it in the four-knot polynomial model with a single-term deletion test using the method “drop1” and Chi-square (Chambers, 1992). This gave insight into whether the background properties such as Country or Dialect should be included as a factor.

The polynomial model estimates the data points between the actual measurements, thereby enabling the calculation of the perceptual thresholds. In this way, we calculated how many milliseconds it took for the subjects to shift perception from an L tone to an HL tone in the following way: in psychophysical studies, the threshold set for the difference, the JND, is often set to 25% and 75% in listeners’ perception (Myers et al., 2020). When the responses for the HL tone are below 25%, the perception of the stimuli is characterized as L. When the responses of HL tones are above 75%, the perception of the stimuli is characterized as HL. The time between 25% and 75% is the interval of uncertainty. The interval of uncertainty is based on the discrimination accuracy values of 25% and 75%, because these are intermediate between random-choice and perfect-agreement performance of two choices (Myers et al., 2020).

## 3 Results

Table 2 shows the results of the MCA. It presents the contributions in percentages (summing up to 100) to the variation in *Dimension 1* of the speaker characteristics by level. The speaker characteristic of coming from South Sudan is the most influential property to *Dimension 1* (nearly 35%). For the mother tongue of the participant’s Parents, both the neighboring language Dinka and the South Sudanese dialect Lou contributed to 17% to the variability. Whether their Parents’ dialect was Panguak, Gajiök, or the same as the participant, contributed very little to the variation (between 2.6% and 0.06%). Concerning the Community of the participants, Gajiök was the largest contributor to the variation with 8%. Whether a participant came from a rural area or from town contributed little to the variation. As for gender, Female contributed nearly 0.4% to variation. Note, however, that only two of the participants were female.

**Table 2.** The Contributions (in Percentage) of Individual Levels of the Qualitative Properties of Participants to Variation.

Variable levels	*Dimension 1*
Community: Gaguang	0.458
Community: Gajaak	3.781
Community: Gajiök	8.158
Community: Lou	0.458
Community: Thien	0.153
Parents: Dinka	17.495
Parents: Gajiök	0.686
Parents: Lou	17.495
Parents: Pangak	1.043
Parents: Same	2.699
Country: Ethiopia	5.148
Country: Ethiopian-South Sudanese border	0.089
Country: South Sudan	34.991
Origin: Rural area	2.303
Origin: Town	4.607
Sex: Female	0.399
Sex: Male	0.0288

The significance of *Dimension 1* as a factor was tested with a single-term deletion test. The results of this inferential test are shown in Table 3. They reveal that the contribution of *Dimension 1* to the model is not significant. For this reason, it was not included as a factor in the polynomial model given further below. Instead, the inclusion of Subject as a random factor in the model accounts adequately for variation in the data.

**Table 3.** Results of a Chi-Square Test Evaluating the Significance of the MCA by Single-Term Deletions.

	ResponseTone ~ poly(MS, 4) * Stimulus + dim1.a + (1 | Listener)			
	Npar	AIC	LRT	Pr(Chi)
<none>		1418.8		
*Dimension 1*	1	1417.9	1.106	0.293
poly(MS, 4):Stimulus	8	1476.3	73.491	9.885e–13***

*p < .05, **p < .01, ***p < .001.

**Figure 5.**
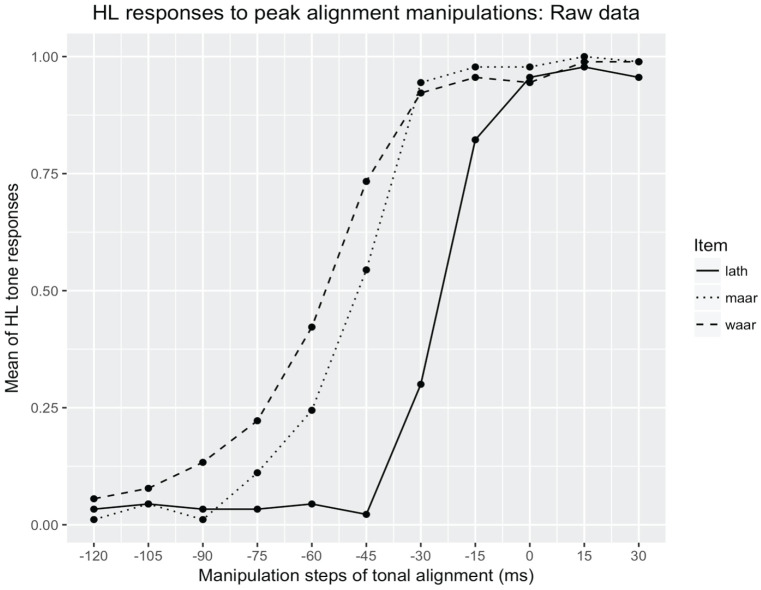
Percentage of HL responses as a function to F0 alignment manipulations (ms).

Figure 5 displays the mean values as a psychometric curve. For each of the three alignment continua or items, this figure shows the percentage of HL responses at each manipulation step, averaged across all listeners. While the experiment collected the responses to stimuli that vary in the alignment of the beginning of the F0 drop from −120 ms before the start of the vowel (early alignment/L tone) to 30 ms into the vowel (late alignment/HL tone), the change from L to HL responses turns out to be abrupt, taking place in the central region within this range. All three items have a steep curve, but the item *la θ* stands out in being particularly steep. In addition, the items differ in the time point on the *x*-axis where crossover from L to HL responses happen.

Figure 5 shows that the percentage of HL responses for item *la θ* is very low (below 7%) until −45 ms before the start of the vowel. From this point, the HL responses increase abruptly. For the other two items, the curve for the HL responses starts rising earlier, and it is slightly less steep, compared with the curve of *la θ *.

The results of the polynomial regression model are presented in Table 4. The main effect of *F0 Alignment* on *Response tone* was significant for all four breaking points: poly(Alignment, 1–4); *p* <.001. The intercept for the model was the stimulus *la θ *. The interactions between *la θ* and *waːr*, and between *la θ* and *maːr* were both highly significant (*p* <.001). These interactions are also shown for each breaking point. The interaction between *la θ* and *maːr* is significant for all breaking points except 3. The interaction between *la* θ and *waːr* is only significant for breaking points 2 and 4.

**Table 4.** Generalized Linear Mixed Model Fit by Maximum Likelihood: Polynomial Model With 4 Knots.

Formula: ResponseTone ~ poly(Align, 4) * Stimulus + (1 | Listener)				
Fixed effects:	Estim.	SE	*z*	Pr (>|*z*|)
(Intercept = *la* θ )	−0.90	0.24	−3.71	0.000205***
poly(Alignm, 4)1	169.71	9.79	17.33	<2e–16***
poly(Alignm, 4)2	53.11	8.78	6.04	1.46e–09***
poly(Alignm, 4)3	−45.26	8.65	−5.22	1.71e–07***
poly(Alignm, 4)4	−48.49	9.15	−5.30	1.16e–07***
Stimulus *laθ*/*maːr*	1.58	0.28	5.55	2.77e–08***
Stimulus *laθ*/*waːr*	1.81	0.22	8.13	4.07e–16***
poly(Alignm, 4)1:Stimulus *la* θ /*maːr*	42.21	17.63	2.39	0.016700*
poly(Alignm, 4)2:Stimulus *laθ*/maːr	−59.62	14.99	−3.97	6.99e–05***
poly(Alignm, 4)3:Stimulus *laθ*/*maːr*	−7.47	13.73	−0.54	0.586272
poly(Alignm, 4)4:Stimulus *laθ*/*maːr*	32.26	14.83	2.17	0.029573*
poly(Alignm, 4)1:Stimulus *la* θ /*waːr*	−10.56	14.14	−0.74	0.455009
poly(Alignm, 4)2:Stimulus *laθ*/*waːr*	−60.87	13.08	−4.65	3.28e–06***
poly(Alignm, 4)3:Stimulus *la* θ /*waːr*	19.10	12.19	1.56	0.117143
poly(Alignm, 4)4:Stimulus *laθ*/*wa:r*	52.47	12.38	4.23	2.25e–05***
(Intercept = *maːr*)	0.67	0.29	2.26	0.02339*
Stimulus *maːr*/*waːr*	0.23	0.28	0.82	0.40793

*p < .05, **p < .01, ***p < .001.

To examine the difference between *maːr* and *waːr*, these interactions were also tested with *maːr* as intercept. As seen from Table 4 (bottom row), the interaction between *maːr* and *waːr* was not significant (*p* = .40). These results confirm the state of affairs observed in Figure 5, where the curves for items *maːr* and *waːr* have a similar shape.

**Figure 6.**
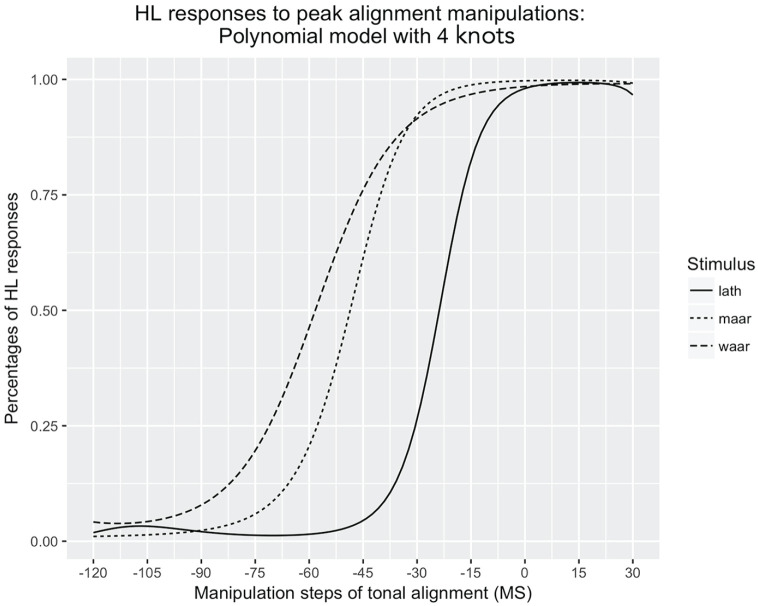
Effect plot of the polynomial model (see Table 4): The percentage of HL responses as a function of F0 alignment manipulations (ms).

Before conducting the polynomial model, it was tested how many knots were needed for the regression. A polynomial regression model with four knots was compared with one with three knots in ANOVA. The results in Table 5 justifies the choice of four knots: compared with three knots, the model with four knots is significantly better (*p* < .001). We did not increase the number of knots to five, because this results in an oversmoothing of the data. The four-knot polynomial functions of the model in Table 4 are plotted in Figure 6.

**Table 5.** Results of a Chi-Square Test Carried Out to Evaluate Two Models: Polynomial with Three Knots Versus Four Knots.

Models	*df*	AIC	BIC	logLik	*SD*	χ^2^	χdf	Pr (>χ^2^)
poly3	13	1443	1521	−708	1417			
poly4	16	1417	1513	−692	1385	32	3	5.2e–07***

*p < .05, **p < .01, ***p < .001.

The polynomial functions allow us to calculate perceptual thresholds for each of the three items. These perceptual thresholds are shown in Table 6. We set the threshold for reliable L tone perception at 25% HL tone perceptions. In the case of *la θ *, this was obtained at −30 ms relative to the boundary between onset and nucleus. For the other two items, 25% of HL responses were located at −57 ms for *maːr* and at −71 ms for *waːr*. We set the threshold for reliable perception of HL tone at 75% HL tone perceptions. For stimulus *la θ *, this boundary was obtained at −17 ms. This differs substantially from the other two items. Here, the same boundary was obtained at −40 ms (*maːr*) and at −45 ms (*waːr*).

**Table 6.** Perceptual Threshold of Tone Alignment: The Amount of Milliseconds it Took for Participants to Shift From a 25% Response HL Identification to 75% Response of HL Identification.

Percentage of HL responses				
25%	75%	75%–25%	(80%–20%)	Stimulus
Mostly L	Mostly HL	Time of uncertainty		
−30.4 ms	−17.4 ms	13.0 ms	(16.3 ms)	*laθ*
−57.7 ms	−40.2 ms	17.4 ms	(22.3 ms)	*ma:r*
−71.1 ms	−45.6 ms	25.5 ms	(32.3 ms)	*wa:r*
−53.0 ms	−34.4 ms	18.6 ms	(23.6 ms)	*M*
20.7 ms	14.9 ms	6.3 ms	(8.0 ms)	*SD*

Expressed as a proportion of the onset domain, the 25% and 75% sensitivity threshold points for the *la θ* set are at approximately 50% (L) and 80% (HL) of onset duration. For the *maːr* and *waːr* sets, these thresholds are located at roughly 20% and 50% of the duration of the onset. Given that a falling contour whose initial turning point is aligned halfway through the onset consonant is reliably perceived as L in the *la θ* set but as HL in the case of the other two sets, the timing of the turning point cannot be the only factor determining classification. We will consider the implications of these findings in section 4.

The time of uncertainty, that is, the time it took for the listeners to shift from 25% to 75% was 13 ms for item *la θ *, 17 ms for item *maːr* and 25 ms for item *waːr*. The mean of these values is 19 ms with an *SD* of 6 ms. In comparison, setting the uncertainty interval more conservatively, at 80%–20%, results in values of 16 ms for the item *la θ *, 22 ms for *maːr*, and 32 ms for *waːr*.

## 4 Discussion

The most striking finding resulting from this perception experiment is how sensitive the Nuer listeners are to variability in tonal alignment. As noted above, we can examine this sensitivity through the uncertainty interval, which is quantified as the time difference between the point where 75% of the stimuli are identified as L-toned, and the point where 75% of the stimuli are identified as HL-toned. The sensitivity is highest for the *la θ* item. Averaged over 30 subjects, the uncertainty interval is only 13 ms wide for this item. This is less than a single 15 ms step on the continuum of manipulations. For the other two items, the uncertainty interval is wider, but still very small: just 17 ms in the case of the *maːr* item, and 25 ms in the case of the *waːr* item. Averaged over the three items, the uncertainty interval stands at 19 ms. This is comparable to the uncertainty interval of 22 ms calculated on the basis of the results in Gårding et al. (1986) for the contrast between the Low (Tone 3) and the Fall (Tone 4) in Mandarin.

The JND values of the current study and of Gårding et al. (1986) are substantially lower than the average JND reported in House (2004). We attribute this discrepancy to the difference in the functional load of tonal timing between these languages. Languages which have tonal contrasts mainly at the utterance level, such as Dutch or English, have a sparse specification, to the effect that the F0 patterns will differ substantially in their overall shape. The same goes for Norwegian, a sparsely specified tone language. As noted earlier, Kelly and Smiljanić (2018) concluded that the height of the tone targets rather than their timing distinguishes the categories from one another.

Considering the differences between the three sets, the results diverge substantially between the *la θ* set on one hand and the *maːr* and *waːr* sets on the other. As seen from Figures 5 and 6, the crossover is located about two steps on the continuum (i.e., 30 ms) earlier for the latter two sets. This difference is reflected in the results of the inferential test, the generalized linear model: *la θ* is significantly different from both *maːr* and *waːr*, whereas the latter do not differ significantly from one another. In addition, there is also the above-mentioned variation in the width of the uncertainty interval: the uncertainty interval is smallest for the *la θ* set (13 ms), and greatest for the *waːr* set (25 ms).

These differences in the location of the crossover and the width of the uncertainty interval offer insights into the factors that play a role in the perception of tonal alignment. In the part of the stimuli that was subject to manipulation, the crucial difference is phonological vowel length, which conditions a substantial difference in vowel duration. The vowel duration in *waːr* and *maːr* are more than 2.5 times longer, at 242 and 224 ms, respectively, than the vowel duration in *la θ *, which stands at 90 ms. Hence, a shift of 15 ms in tonal alignment in the manipulations will condition a proportionally greater difference in the melodic pattern over the vowel if the vowel is short than when the vowel is overlong. This matters, because the vowel is the most salient part of the syllable. In the *la θ* item, a 15 ms shift in the alignment of the F0 drop corresponds to 16% of the entire vocalic domain. In *waːr* and *maːr*, in contrast, a 15 ms shift in F0 alignment affects 6%–7% of the vowel: 6.1% in the case of *waːr*, and 6.6% in the case of *maːr*. This proportional difference helps to explain why the uncertainty interval is smaller for *la θ *, which has a short vowel, than for *waːr* and *maːr*, which have an overlong vowel. The observed role of vowel duration is in line with the perception study by Franich (2016), who found that vowel duration is a significant factor in distinguishing between contour and level tones.

The variability in the location of the uncertainty interval can also be explained with reference to the impact of the manipulations on the vocalic domain. While the size of the drop was fixed (5 ST), the steepness mirrored the tilt in the natural data on which the stimuli were based. As seen from Figure 3, the tilt is steeper in the realizations of the members of the *la θ* minimal pair than in those of the other two pairs. In the manipulations, shown in Figure 4, the steeper slope in the *la θ* item has the effect that the early aligned manipulations reach the endpoint of the F0 drop before the beginning of the vowel, whereas the early aligned manipulations of the *maːr* and *waːr* items already have the final part of the drop realized in the vocalic domain. Concretely, the sixth point on the continuum, where the F0 drop starts at 45 ms before the beginning of the vowel, represents a manipulation in which the F0 drop already affects the vocalic domain sufficiently to make the participants perceive the majority of *maːr* and *waːr* stimuli as Fall-toned members. In the case of *la θ *, the stimulus with the same alignment has the F0 drop only affecting a small part of the vocalic domain. Evidently, what matters for the perception of tonal alignment is not the timing of the start of the F0 drop within the onset consonant, but the impact of the F0 movement on the vocalic domain.

It makes sense for the timing of the start of the drop to be less relevant than that of the impact of the resulting contour on the vocalic domain: depending on the segmental composition of the syllable, the syllable onset is often voiceless (e.g., [p]), and then the beginning of the F0 drop is not phonetically realized. In other words, when it comes to the alignment of an F0 change conditioned by an initial turning point in the syllable onset, its impact on the vocalic domain represents a more consistent phonetic phenomenon than the alignment of the initial turning point within the onset in itself.

The fact that that the location of the category boundary is significantly later for the *la θ* continuum—where the vowel is short and the slope steeper—than for the other two continua, which present a long vowel and a shallower slope can be understood in relation to earlier work on factors that affect the perception of F0 pattern in addition to alignment (Barnes et al., 2021; D’Imperio, 2000; D’Imperio & House, 1997; Niebuhr, 2003; Niebuhr et al., 2011). The fact that these factors weigh in on the perception of melodic patterns lends support to the interpretation that the hearer integrates a range of properties. This perspective forms the basis of the Tonal Center of Gravity approach (Barnes et al., 2012, 2021). In recent work (Barnes et al., 2021), this approach has been developed so as to take into account the metrical prominence of syllables, and Barnes and colleagues (Barnes et al., 2021, p. 18) are working toward developing the TCoG measure so that it can take into account differences in sonority of constituents within the syllable.

Our finding that the item with a steeper F0 slope yields a sharper crossover in the perception study matches up with earlier work on Mandarin. In a perception experiment investigating the perception of the contrast between the Mandarin Rise (Tone 2) and Low (Tone 3) in the citation forms, Shen and Lin (1991) created two continua, which differed in the size of F0 changes, while the time domain over which these changes were realized was kept constant. As a result, the F0 changes were steeper in the continuum involving greater changes. In the responses, the uncertainty interval was narrower (i.e., the crossover was steeper) for the continuum involving greater F0 changes.

Finally, the results of our perception study call into question an influential hypothesis on the phonetic realization of contour tones. House (1990, pp. 133–134) hypothesized that, to be optimally perceived as a falling contour, the turning point that defines the beginning of the F0 movement needs to be aligned 30–50 ms into the vowel. House motivated this hypothesis on the basis of the reduced perceptibility of pitch contrast at the beginning of the vowel, where the formants are affected by consonant-induced transitions. However, evidence from both speech production and from speech perception presented in this paper indicates that the initial turning point of the Nuer HL is aligned considerably earlier: at the boundary between the onset consonant and the vowel. This shows that Nuer listeners are paying attention to what happens at the beginning of the syllable, contrary to the analysis of House (1990).

## 5 Conclusion

In conclusion, the perception of changes in tonal alignment by speakers of Nuer is very fine-grained: averaged across three items, the 25%–75% uncertainty interval is only 19 ms wide. Just like Gårding et al. (1986), we find this sensitivity in relation to the contrast between a Low tone and an early aligned Fall (patterns A and B in Figure 1). Against the background of earlier work on JND in relation to F0, this is a surprisingly low threshold for listeners to hear a difference between two categories. On the basis of languages where the specification for tone is either intonational or sparsely specified at the word level, House (2004) concluded that it takes at least 50 ms for two tonal shapes that differ solely in F0 alignment to be distinguished reliably. We attribute the fact that the sensitivity is substantially greater in Mandarin and in Nuer to the fact that these are languages in which tone is densely specified at the word level.

The variation between items in both the location of the perceptual crossover and in its steepness supports the interpretation that identification is determined by the perceptual impact of the F0 change on the vowel, the syllable nucleus. This chimes with recent work showing that phonetic parameters other than F0 alignment are integrated with the latter in the perception of tone (Barnes et al., 2021; Niebuhr et al., 2011). For this reason, the contrast between Low and Falling tones in Nuer can be of interest in the development of Tonal Center of Gravity, a holistic model in which F0 alignment is integrated with other properties of the tonal pattern and segmental material on which it is realized.

Finally, the timing differences observed, that is, of 13, 17, and 25 ms, are relevant to the design of future investigations into tonal alignment in languages in which this parameter has a high functional load. The step size of the manipulations should be small enough to offer a clear resolution of the perceptual sensitivity as fine-grained as this.

## Notes

1. In this environment the realization of the Low (Tone 3) does not include a late high target.

2. In a perception experiment involving a choice between two options, the interval of uncertainty can be defined as the time range between 25% and 75% selections of either of the two options (Myers et al., 2020).

3. See Gjersøe (2019) for further details, including results on H-toned items.

4. The slope of the nuclei was measured by extracting F0 at 1/10th of the vowel and at 9/10th of the vowel. The initial point was subtracted from the final point of each target.

5. The meaning of the sentence in section 2.1 may refer to an event of shaking such as, for example, an earthquake. The fricative [*θ*] corresponds to the phoneme /t/ and is realized in a coda position.

6. These items differ greatly in vowel duration and it is interesting to note that items with a short vowel such as *lâ* θ realize a fall of this size: 5 ST over 90 ms. Xu and Sun (2002) found that speakers need on average 124 ms to realize a falling contour tone of 4 ST. Although their study cannot be compared directly with this one, it suggests that the maximum speed of F0 change for some speakers is higher than an average of 124 ms to realize a fall of 4 ST.
